# Enhancing Model Generalizability in Medical Artificial Intelligence: Systematic Comparison of Categorical Encoding and Sampling Techniques for Imbalanced Data

**DOI:** 10.2196/75655

**Published:** 2026-04-13

**Authors:** Chien-wei Chuang, Chung-Kuan Wu, Chao-Hsin Wu, Ben-Chang Shia, Mingchih Chen

**Affiliations:** 1 Graduate Institute of Business Administration, Fu Jen Catholic University, 510 Zhongzheng Rd, Xinzhuang District, New Taipei City, 242062, Taiwan, 886 2905-3895; 2 Artificial Intelligence Development Center, Fu Jen Catholic University, New Taipei City, Taiwan; 3 Division of Nephrology, Shin Kong Wu Ho-Su Memorial Hospital, Taipei, Taiwan; 4 Dialysis Access Management Center, Shin Kong Wu Ho-Su Memorial Hospital, Taipei, Taiwan; 5 School of Medicine, Fu Jen Catholic University, New Taipei City, Taiwan; 6 Division of Digital Informatics Management, Department of Digital Medicine, Shin Kong Wu Ho-Su Memorial Hospital, , Taipei, Taiwan

**Keywords:** machine learning, data preprocessing, clinical prediction models, medical informatics, feature engineering

## Abstract

**Background:**

Despite the increasing use of machine learning (ML) in clinical research, the early stages of data preparation, especially for structured clinical data, often receive limited methodological scrutiny. These datasets typically contain missing values, complex categorical variables, and imbalanced class distributions, all of which complicate downstream model development and interpretation.

**Objective:**

This study introduces a structured preprocessing framework designed to address common challenges in medical tabular data and to assess how preprocessing choices affect the stability and portability of predictive models across settings.

**Methods:**

We constructed a modular workflow comprising 3 components. First, preprocessing strategies included imputation for missing data, 3 types of categorical encoding (one-hot, frequency, and target), and resampling approaches for class imbalance (Synthetic Minority Over-sampling Technique [SMOTE] and Random Over Sampling Example [ROSE]). Second, 6 classification algorithms were used to evaluate performance patterns, including logistic regression (LGR), decision tree (DT), random forest, XGBoost (XGB), CatBoost (CAT), and light gradient-boosting machine (LightGBM). Third, we assessed cross-dataset portability using 2 datasets with distinct data-generating mechanisms: a registry for patients with end-stage renal disease (ESRD; n=412) and the population-based Behavioral Risk Factor Surveillance System (BRFSS) 2015 survey. For each dataset, we independently cleaned, standardized, encoded, tuned, and evaluated models using the same predefined hyperparameter search space, without cross-dataset feature matching or pooling the area under the ROC curve (AUC) calculations; the complete pipeline was then rerun on BRFSS as an external replication.

**Results:**

One-hot encoding in combination with ROSE yielded the most consistent performance improvements in terms of AUC (0.940) and accuracy (0.932), particularly for classifiers sensitive to class distribution. Notably, ROSE enhanced sensitivity without substantially distorting the original data structure. Feature importance rankings also contributed to model interpretability, and performance trends were largely reproducible in cross-context application.

**Conclusions:**

Our findings suggest that preprocessing decisions often treated as ancillary play a central role in shaping model outcomes, especially in high-variance clinical datasets. The proposed framework offers a reproducible and adaptable tool for aligning data preparation with the unique demands of health care prediction tasks and may serve as a foundation for future efforts to standardize preprocessing in clinical ML workflows.

## Introduction

Although health care institutions generate vast volumes of health and clinical tabular data on a daily basis, these valuable resources frequently encounter significant bottlenecks when being translated into effective predictive models [1,2]. In practice, suboptimal model performance is often not attributable to the limitations of the algorithms themselves but rather to the lack of consistency and strategic planning in the data preprocessing pipeline [3,4]. Prior studies have demonstrated that various preprocessing techniques such as data imputation, normalization, and feature selection are highly sensitive in their impact on model performance [5,6], and may even introduce bias or lead to overfitting [7]. Consequently, a critical yet insufficiently addressed question arises: Can we establish a generalizable and reproducible data processing framework that assists users in selecting appropriate machine learning (ML) predictive models?

In the domain of medical informatics, ML models have been extensively used for predictive analyses of health and clinical data. However, extant literature has predominantly focused on comparing algorithmic accuracy and model performance [8,9], while offering limited systematic investigation into critical data processing steps such as data cleaning, feature encoding, and handling of class imbalance [10,11]. This oversight is particularly consequential given the inherent characteristics of medical datasets, which frequently exhibit high rates of missing values, numerous categorical variables, and severely imbalanced outcome distributions—factors that significantly influence predictive outcomes depending on the preprocessing choices made [12,13].

Despite the enormous volume of clinical and health data generated daily by health care institutions, transforming these data into effective predictive models remains challenging due to pervasive issues such as high missingness, class imbalance, and the need for robust variable encoding. Although prior studies have examined individual procedures such as comparing one-hot versus target encoding [14,15] and evaluating synthetic data augmentation techniques like the Synthetic Minority Over-sampling Technique (SMOTE) [16], systematic evaluations of the interactive effects among diverse preprocessing workflows are exceedingly scarce, particularly across heterogeneous medical datasets. Furthermore, there is a notable gap in comparative research on encoding strategies (eg, one-hot, frequency, and target encoding) and imbalance correction methods (eg, SMOTE and Random Over Sampling Example [ROSE]), which hampers the establishment of best practices in clinical ML.

This study proposes a comprehensive, reproducible framework specifically for medical tabular data. The framework is built upon three core components: (1) the Data Processing Strategy Layer, which systematically evaluates essential preprocessing techniques, including missing value imputation, variable encoding, and class imbalance correction; (2) the Model Selection and Optimization Layer, which ensures compatibility with a diverse range of supervised learning algorithms; and (3) Cross-Dataset Validation, which tests the framework’s transferability and consistency on 2 highly heterogeneous real-world clinical datasets. This design not only streamlines the preprocessing pipeline but also minimizes overlap with subsequent methodological details.

The primary contribution of this work lies in developing and empirically validating an end-to-end data processing framework that transcends the limitations of single-model, single-dataset analyses. By shifting the focus from solely model-centric performance metrics to a holistic methodological architecture, our approach provides both data scientists and clinical researchers with a modular and standardized workflow. This framework is expected to bridge the gap between algorithm development and clinical application, offering robust empirical evidence and actionable guidance for advancing predictive modeling in health care.

## Methods

### Data Collection

**Figure 1.**
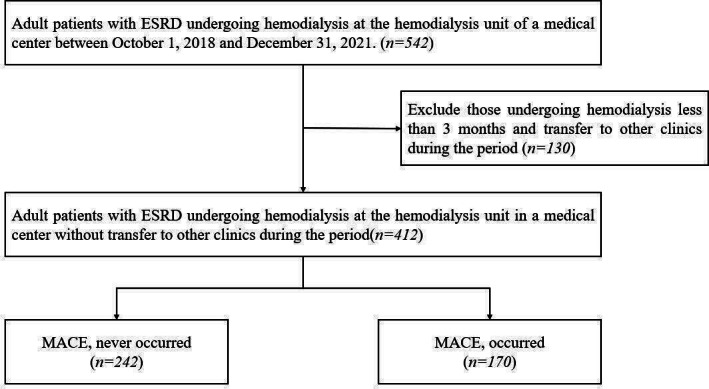
Flowchart of study population selection and major adverse cardiovascular event (MACE) grouping. ESRD: end-stage renal disease; MACE: major adverse cardiovascular event;

Initially, we enrolled 542 adult patients with ESRD undergoing hemodialysis at the hemodialysis unit of a medical center between October 1, 2018, and December 31, 2021. Patients who had received hemodialysis for less than 3 months or who were transferred to other clinics during the study period were excluded. After these exclusions, 412 adult patients with ESRD undergoing chronic hemodialysis without transfer remained eligible for analysis. Among them, 242 patients had no occurrence of major adverse cardiovascular events (MACEs), while 170 experienced at least one MACE. The primary objective of this study was to determine the incidence of MACEs in this population. A flowchart of the study participants is presented in Figure 1. The detailed baseline demographic and clinical characteristics of the cohort are summarized in Table 1.

**Table 1.** Initial demographic and clinical profiles of the research cohort.

Variables	Overall (n=412)	MACE^a^		*P* value
		Never occurred (n=242)	Occurred (n=170)	
Age, mean (SD)	69.19 (12.14)	67.96 (12.59)	70.94 (11.29)	.01
Sex (female), n (%)	192 (46.6)	122 (50.4)	70 (41.2)	.08
AVG (Arteriovenous graft), n (%)	57 (13.8)	31 (12.8)	26 (15.3)	.57
AV cal, n (%)^b^	196 (62.6)	106 (56.1)	90 (72.6)	.005
AR^c^, n (%)				.14
None	178 (43.2)	115 (47.5)	63 (37.1)
Negligible or mild	125 (30.3)	68 (28.1)	57 (33.5)
Moderate	8 (1.9)	3 (1.2)	5 (2.9)
AS, n (%)^d^				.03
-	101 (24.5)	56 (23.1)	45 (26.5)
None	277 (67.2)	172 (71.1)	105 (61.8)
Negligible or mild	22 (5.3)	12 (5.0)	10 (5.9)
Moderate	11 (2.7)	2 (0.8)	9 (5.3)
Severe	1 (0.2)	0 (0.0)	1 (0.6)
LVH type, n (%)^e^				.02
1	131 (43.8)	72 (39.8)	59 (50.0)
2	106 (35.5)	63 (34.8)	43 (36.4)
3	28 (9.4)	24 (13.3)	4 (3.4)
4	34 (11.4)	22 (12.2)	12 (10.2)
Comorbidities, n (%)				
DM^f^	198 (48.1)	99 (40.9)	99 (58.2)	.001
Dyslipid	220 (53.4)	130 (53.7)	90 (52.9)	.96
PAOD^g^	111 (26.9)	53 (21.9)	58 (34.1)	.01
Medication, n (%)				
Insulin	85 (20.6)	33 (13.6)	52 (30.6)	<.001
Statin	137 (33.3)	73 (30.2)	64 (37.6)	.14
Antiplatelet	199 (48.3)	82 (33.9)	117 (68.8)	<.001
Calcitriol	173 (42.0)	104 (43.0)	69 (40.6)	.70
No of hypotension episodes, mean (SD)	5.41 (3.26)	5.46 (3.21)	5.34 (3.33)	.71
CXR_AoAC, n (%)^h^				<.001
0	120 (31.4)	90 (40.5)	30 (18.8)
1	83 (21.7)	49 (22.1)	34 (21.2)
2	107 (28.0)	49 (22.1)	58 (36.2)
3	72 (18.8)	34 (15.3)	38 (23.8)

a MACE: major adverse cardiovascular event.

b AV cal: aortic valve calculation

c AR: aortic regurgitation.

d AS: aortic stenosis.

e LVH: left ventricular hypertrophy.

f DM: diabetes mellitus.

g PAOD: peripheral arterial occlusion disease.

h CXR_AoAC: chest X-ray for aortic arch calcification.

This study specifically targeted the unique clinical needs and complexities of patients with ESRD by collecting 84 variables. These variables were meticulously selected based on their significant impact on clinical outcomes in patients with ESRD. Demographic data, such as age and gender, were included, and dialysis vintage reflected the duration and history of each patient’s dialysis treatment. Additionally, we detailed the anatomical and functional characteristics of the aortic and mitral valves, which are crucial for understanding cardiovascular complications in patients with ESRD. Specifically, we included parameters such as types of arteriovenous access (AVA), mitral valve calcification (MV calc), aortic regurgitation (AR), aortic stenosis (AS), mitral regurgitation (MR), and mitral stenosis (MS).

In terms of cardiovascular health, this study placed particular emphasis on the grading and types of left ventricular hypertrophy (LVH) [17] and the ejection fraction (EF) of the heart, which are critical indicators of cardiovascular health in patients with ESRD. Given the high prevalence and impact of comorbidities such as diabetes mellitus, hypertension, dyslipidemia, coronary artery disease, heart failure, chronic obstructive pulmonary disease, liver cirrhosis, malignancy, arrhythmia, and a history of amputation among patients with ESRD, we included these comorbidities in our analysis.

To better meet the clinical needs of patients with ESRD, we expanded the range of biochemical laboratory data. This included comprehensive assessments of total protein, albumin, liver enzymes (aspartate aminotransferase and alanine aminotransferase), alkaline phosphatase [18], total bilirubin, lipid profiles, glucose levels, complete blood count, iron studies, aluminum levels, postdialysis weight, uric acid, and key electrolytes. To address the specific needs of patients with ESRD, we further measured calcium and phosphate metabolism indicators, such as calcium and phosphate levels, urea kinetics (Kt/V), parathyroid hormone levels, and the calcium-phosphate product, to more effectively manage mineral and bone disorders in these patients.

The medication history was also thoroughly documented, particularly focusing on drugs commonly used in the management of ESRD, such as phosphate binders, calcitriol, and other treatments relevant to the patients’ condition.

This study included 412 patients. The mean age was 69.19 years, with older patients more likely to experience MACE (70.94 years vs 67.96 years, *P*=.01). Females comprised 46.6% of the cohort, with a lower proportion in the MACE group, although this difference was not statistically significant (*P*=.08). Aortic valve calcification was more prevalent in the MACE group (72.6% vs 56.1%, *P*=.005), and AS as well as certain types of LVH were also associated with the occurrence of MACE. Diabetes mellitus (DM) and peripheral arterial occlusive disease (PAOD) were more common among patients who experienced MACE (DM: 58.2% vs 40.9%; *P*=.001; PAOD: 34.1% vs 21.9%; *P*=.008). In terms of medication use, a higher proportion of patients in the MACE group were on insulin (*P*<.001) and antiplatelet drugs (*P*<.001). These results provide an overview of the baseline characteristics of the patients and offer important references for improving the accuracy of predictive models.

### Data Preparation

**Figure 2.**
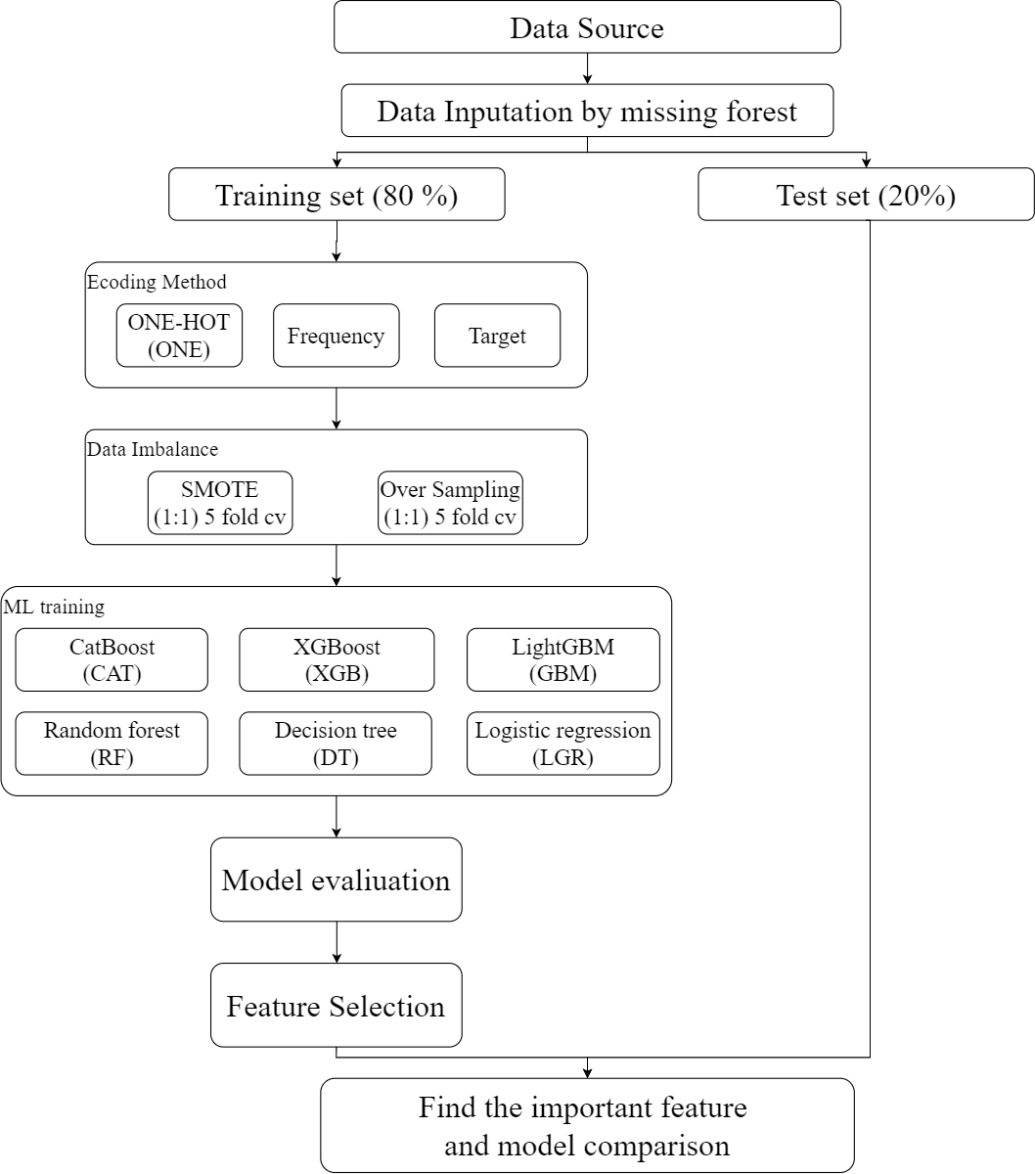
Research analysis process framework diagram. Preprocessing components are fit on training folds only and then applied to validation folds; ROSE or SMOTE are applied to training folds only to prevent leakage.

The data preprocessing methodology in this study is organized into 2 primary components: variable encoding and data balancing. The overall analytical workflow and preprocessing framework are illustrated in Figure 2. This integrated approach is designed to enhance the quality and representativeness of clinical datasets, thereby improving the robustness and generalizability of subsequent predictive models.

### Data Imputation

Missing data are a common challenge in clinical datasets and can significantly compromise the validity of statistical inferences if not appropriately addressed. In this study, we used a nonparametric multiple imputation strategy using the *missForest* package in R (R Core Team) [19]. This method uses random forest models to iteratively predict missing values based on observed data, effectively capturing complex nonlinear associations and interactions between variables. Unlike simpler imputation techniques such as mean substitution or k-nearest neighbors, missForest has been shown to yield more accurate and less biased estimates in both continuous and categorical variables, particularly in mixed type medical data [15,20]. This approach offers a robust and flexible foundation for downstream ML analysis while preserving the integrity of the original dataset.

Compared to SMOTE, which generates new samples through linear interpolation between neighboring minority class points, ROSE applies a smoothed bootstrap technique. It estimates a kernel density around each minority instance and samples new points from this local distribution. This approach allows ROSE to preserve the original variance and nonlinear structure of the data, making it particularly effective for clinical datasets where preserving subtle distributional patterns is important.

Imputation models were fit on the training fold only and then applied to the corresponding validation fold within each split. No statistics from the validation fold were used to fit the imputation model. This fold-wise procedure was repeated across all folds to prevent information leakage.

### Variable Encoding and Expansion

In clinical datasets, categorical variables are abundant and require conversion into numerical formats to be effectively used in statistical and ML models. Three encoding methods were implemented to address this challenge, each offering a unique balance between preserving information and managing computational complexity. Although all 3 approaches aim to transform qualitative data into quantitative representations, they differ in their operational mechanisms and associated trade-offs.

One-hot encoding converts each categorical variable into a series of binary indicators, where each category is represented by an individual binary feature. This method maintains the inherent nonordinal nature of the original variable but can lead to a substantial increase in dimensionality, particularly when the variable in question has many unique categories. In contrast, target encoding substitutes each category with a statistical summary such as the mean, weighted mean, or smoothed mean of the target variable computed from the training dataset. This not only reduces dimensionality but also encapsulates the predictive relationship between the categorical feature and the outcome variable, although it necessitates careful handling to avoid target leakage [14]. A third approach, frequency encoding, assigns to each category a numerical value based on its relative frequency within the dataset. This method is highly efficient in reducing computational burden and memory usage, as it compresses categorical information into a single continuous variable without imposing any artificial order [21].

To combine the complementary strengths of these methods, a unified encoding strategy was adopted. Specifically, the one_hot function from the *mltools* package in R was applied to perform one-hot encoding, which expanded the original set of 83 variables to 113. This expansion increased the granularity of the dataset, facilitating a more detailed representation of the clinical phenomena under this research. The inclusion of target and frequency encoding provided an additional layer of comparison, enabling an evaluation of their relative performance under conditions of significant missingness and class imbalance. Prior work has demonstrated that, particularly in datasets with high proportions of missing data and imbalanced classes, preprocessing methods based on one-hot encoding can significantly enhance both accuracy and robustness in classification tasks [15].

All categorical encoders were fit on the training fold only and then applied to its validation fold. Target encoding used an out-of-fold smoothing scheme: for each fold, category means and smoothing weights were computed from the training fold and then mapped to the validation fold. No target information from the validation fold was used to compute encodings.

### Data Imbalance

Clinical datasets often exhibit imbalanced class distributions, where the minority class, despite its clinical significance, is underrepresented. Such imbalance can lead to biased models that disproportionately favor the majority class. To counteract this, 2 complementary strategies, Random Over-Sampling (ROS) and the SMOTE, were incorporated, along with an evaluation of the ROSE method [22].

The ROSE method uses a bootstrap resampling framework augmented by kernel density estimation to generate synthetic samples for the minority class. This approach avoids the pitfalls associated with simply duplicating minority samples, offering a more nuanced correction of the class distribution. Menardi and Torelli [23] provide an extensive discussion of these resampling techniques, emphasizing their utility in balancing datasets for binary classification tasks. On the other hand, SMOTE, as introduced by Chawla et al [24], synthesizes new minority class instances by interpolating between existing samples. This technique enriches the minority class by generating additional, diverse examples, thereby improving the model’s ability to capture the characteristics of rare events. Empirical studies have shown that SMOTE can lead to marked improvements in performance metrics, such as the area under the ROC curve (AUC), though it may overgeneralize when faced with extreme imbalance.

Comparative evaluations in the literature further underscore the respective merits of these techniques. For instance, Kamalov et al [25] found that ROSE, despite its relative simplicity, remains a stable and computationally efficient solution, especially in multi-label contexts. Similarly, investigations by Gnip et al [26] and Nguyen et al [27] have confirmed that both ROSE and SMOTE are effective in mitigating the adverse effects of class imbalance, with the optimal choice being contingent upon the specific characteristics of the dataset and the available computational resources.

#### Integration of Preprocessing Components

The overall preprocessing workflow was designed to integrate the 2 components variable encoding and data balancing, into a coherent sequence. Initially, missing values were imputed using the random forest approach, ensuring that the dataset was complete and reliable. This was followed by the transformation of categorical variables via the combined encoding strategy, which not only translated qualitative data into numerical features but also expanded the feature set to enhance data granularity. Finally, class imbalance was addressed through the application of ROSE and SMOTE, thereby ensuring that the resulting dataset was well-suited for the development of predictive models.

Each preprocessing step was carefully implemented so that subsequent operations built upon an increasingly refined version of the dataset. The robust imputation stage preserved the original data’s distributional properties, while the encoding procedures facilitated the construction of a rich, multidimensional feature space. The balancing techniques further adjusted the dataset to prevent bias toward the majority class, enabling the models to more effectively capture the subtleties of clinically significant, albeit rare, events.

#### Methodological Rationale and Literature Justification

The methodological choices articulated above are firmly grounded in this body of literature. The use of a random forest–based imputation method is well-supported by studies demonstrating its efficacy in preserving data structure and ensuring the validity of statistical inferences [15,19]. Similarly, the comparative evaluation of encoding methods draws on prior research that highlights the trade-offs between dimensionality, computational efficiency, and the risk of target leakage [14,21]. Moreover, the advantages of one-hot encoding in contexts marked by high missingness and class imbalance have been substantiated by empirical investigations [15]. The incorporation of data balancing strategies, including ROSE and SMOTE, is equally well-documented, with seminal works establishing their effectiveness in correcting class imbalances [23,24], and more recent studies further validating these methods [25-27].

The data preprocessing methodology presented herein represents a rigorous and systematic approach to overcoming the multifaceted challenges inherent in clinical datasets. By sequentially addressing missing data, encoding categorical variables into a more informative numerical format, and correcting class imbalances, the workflow transforms raw clinical data into a format that is both analytically robust and amenable to predictive modeling. This integrated preprocessing pipeline is instrumental in bridging the gap between the complexities of clinical data and the demands of advanced statistical and ML techniques, ultimately contributing to the development of models that are both reliable and clinically pertinent.

Class rebalancing was applied only to the training portion of each fold. Validation folds preserved the original class distribution. For transparency and reproducibility, we report resampling parameters in Table S4 in Multimedia Appendix 1, including SMOTE and ROSE sampling ratios and the random seed used.

### Data Segmentation

k-fold cross-validation is a commonly used resampling technique for evaluating the performance of ML models. Specifically, the dataset is divided into k subsets (folds), and in each iteration, one subset is used as the validation set while the remaining k-1 subsets are used as the training set. This process is repeated k times, with a different subset used for validation each time.

This method effectively prevents overfitting and provides a more robust evaluation of the model. It is particularly useful for estimating the generalization error in small datasets [28].

In this study, we used 5-fold cross-validation to obtain an accurate estimate of model performance through multiple splits and evaluations while reducing bias caused by data partitioning.

### Robust Model Engineering: Tuning, Processing, and Risk Mitigation

All preprocessing and modeling steps, including cleaning, standardization, encoding, resampling, model fitting, and hyperparameter tuning, were executed separately within each dataset. No features, encoders, parameters, or statistics were transferred across datasets. For each model, hyperparameters were tuned by grid search with 5-fold cross-validation on the training set. A single predefined hyperparameter search space and evaluation criterion, detailed in Table S1 in Multimedia Appendix 1, was used for both ESRD and BRFSS. Grid search and model selection were rerun independently in each dataset so that optimal hyperparameters were learned within ESRD and BRFSS separately and were never reused across datasets.

### Model Building and Validation-Phase 1

In Phase 1, we compared alternative encoding and class-imbalance handling strategies for predicting MACE. Traditional logistic regression and 6 ML models were evaluated, including decision trees [29], random forests [30,31], XGBoost [32], CatBoost [33], and LightGBM [34]. All models were trained and evaluated using 5-fold cross-validation [35] within each dataset, following the generic engineering framework described in the previous subsection, that is, grid search hyperparameter tuning with the shared search space in Table S1 in Multimedia Appendix 1 and dataset-specific model selection.

The workflow, including preprocessing, resampling, and hyperparameter tuning, was then rerun independently on the BRFSS 2015 dataset to assess cross-dataset portability of the pipeline rather than to externally validate a single ESRD-trained model. All training was executed on a workstation equipped with an Intel Core i9 10th-generation CPU (3.3 GHz) and 64 GB RAM, using the CPU only. Average runtimes for each pipeline are reported in Tables2  3 to help readers gauge computational cost. In this phase, multiple performance metrics were used, including accuracy, sensitivity, specificity, precision, *F*_1_-score, and AUC. The mean and SD of these metrics were calculated across folds to assess predictive accuracy, robustness, and consistency. The overall workflow is summarized in algorithm S1 in Multimedia Appendix 1.

**Table 2.** Evaluation metrics of 5-fold cross-validation using all encoding methods and imbalanced data processing methods.

Encoding, imbalance, and machine learning method			Accuracy		*F*_1_-score		AUC^a^		Runtime (sec)
			Mean (SD)	95% CI	Mean (SD)	95% CI	Mean (SD)	95% CI
One-hot encoding		
SMOTE^b^		
RF^c^			0.711 (0.048)	0.680‐0.752	0.660 (0.062)	0.611‐0.739	0.708 (0.050)	0.658‐0.757	23.9
CAT^d^			0.706 (0.062)	0.701‐0.751	0.689 (0.038)	0.653‐0.726	0.739 (0.044)	0.695‐0.781	124.0
LightGBM^e^			0.702 (0.043)	0.675‐0.723	0.694 (0.041)	0.666‐0.705	0.713 (0.032)	0.670‐0.764	17.4
ROSE^f^		
RF			0.932 (0.106)^g^	0.783‐1.000	0.917 (0.131)^g^	0.752‐1.000	0.938 (0.117)^g^	0.793‐1.000	23.9**^g^**
CAT			0.918 (0.094)^g^	0.795‐1.000	0.890 (0.131)^g^	0.730‐0.995	0.932 (0.103)^g^	0.788‐1.000	401.0**^g^**
LightGBM			0.932 (0.112)^g^	0.759‐1.000	0.918 (0.137)^g^	0.754‐1.000	0.940 (0.116)^**g**^	0.794‐1.000	19.7**^g^**
Frequency encoding		
SMOTE		
LGR			0.604 (0.094)	0.433‐0.660	0.502 (0.052)	0.188‐0.518	0.565 (0.058)	0.493‐0.582	0.110
CAT			0.619 (0.079)	0.479‐0.646	0.600 (0.033)	0.375‐0.627	0.613 (0.042)	0.498‐0.692	106.0
LightGBM			0.619 (0.102)	[0.321‐0.630]	0.490 (0.086)	[0.304‐0.580]	0.602 (0.069)	0.515‐0.663	15.0
ROSE		
RF			0.896 (0.158)	0.666‐1.000	0.867 (0.206)	0.553‐1.000	0.899 (0.194)	0.623‐1.000	19.7
CAT			0.886 (0.127)	0.678‐1.000	0.864 (0.150)	0.694‐1.000	0.913 (0.140)	0.690‐1.000	329.0
LightGBM			0.870 (0.145)	0.708‐1.000	0.825 (0.213)	0.576‐1.000	0.886 (0.175)	0.707‐1.000	19.9
Target encoding		
SMOTE		
LGR			0.621 (0.044)	0.324‐0.684	0.507 (0.102)	0.213‐0.567	0.576 (0.055)	0.497‐0.587	0.110
CAT			0.648 (0.038)	0.477‐0.649	0.600 (0.053)	0.266‐0.662	0.624 (0.030)	0.492‐0.687	106.0
LightGBM			0.646 (0.031)	0.464‐0.638	0.515 (0.182)	0.242‐0.708	0.605 (0.058)	0.512‐0.671	14.8
ROSE		
RF			0.882 (0.184)	0.565‐1.000	0.866 (0.202)	0.553‐1.000	0.893 (0.204)	0.615‐1.000	19.4
CAT			0.876 (0.131)	0.641‐1.000	0.828 (0.214)	0.651‐1.000	0.900 (0.159)	0.638‐1.000	285.0
LightGBM			0.891 (0.155)	0.669‐1.000	0.850 (0.225)	0.602‐1.000	0.889 (0.178)	0.634‐1.000	19.5

a AUC: area under the ROC curve.

b SMOTE: Synthetic Minority Over-sampling Technique.

c RF: random forest.

d CAT: CatBoost.

e LightGBM: light gradient boosting machine.

f ROSE: Random Over Sampling Examples

g This method has the highest model accuracy and a small standard deviation, indicating that it has a relatively good ability to make judgments

**Table 3.** Evaluation metrics of remodeling with the top 15 most important variables, using one-hot encoding and SMOTE or ROSE.

ETL and machine learning methods		Accuracy		*F*_1_-score		AUC^a^		Runtime (sec)
		mean (SD)	95% CI	mean (SD)	95% CI	mean (SD)	95% CI
SMOTE^b^	
LGR^c^		0.723 (0.045)	0.668‐0.779	0.680 (0.050)	0.618‐0.743	0.728 (0.044)	0.673‐0.783	0.08
DT^d^		0.636 (0.035)	0.592‐0.680	0.547 (0.073)	0.457‐0.637	0.622 (0.044)	0.567‐0.676	2.97
RF^e^		0.699 (0.027)	0.665‐0.733	0.675 (0.027)	0.642‐0.709	0.711 (0.020)	0.687‐0.736	9.79
XGB^f^		0.658 (0.021)	0.632‐0.684	0.630 (0.052)	0.566‐0.694	0.670 (0.024)	0.641‐0.700	2.59
CAT^g^		0.699 (0.044)	0.644‐0.754	0.703 (0.027)	0.669‐0.737	0.737 (0.021)	0.711‐0.763	93.8
LightGBM^h^		0.709 (0.030)	0.671‐0.747	0.682 (0.045)	0.626‐0.738	0.717 (0.034)	0.675‐0.760	14.4
ROSE^i^	
LGR		0.738 (0.030)	0.700‐0.776	0.699 (0.050)	0.636‐0.762	0.755 (0.050)	0.694‐0.817	0.08
DT		0.755 (0.089)	0.644‐0.866	0.703 (0.126)	0.547‐0.860	0.750 (0.100)	0.626‐0.875	2.96
RF		0.891 (0.131)	0.728‐1.000	0.879 (0.133)	0.713‐1.000	0.915 (0.133)^j^	0.750‐1.000	9.05
XGB		0.913 (0.122)^j^	0.762‐1.000	0.899 (0.138)^j^	0.728‐1.000	0.906 (0.137)	0.736‐1.000	3.28
CAT		0.862 (0.073)	0.771‐0.952	0.831 (0.092)	0.717‐0.945	0.896 (0.092)	0.782‐1.000	270**^j^**
LightGBM		0.906 (0.115)^j^	0.763‐1.000	0.892 (0.119**)**^j^	0.745‐1.000	0.925 (0.112)^j^	0.786‐1.000	19.2

a AUC: area under the ROC curve.

b SMOTE: Synthetic Minority Over-sampling Technique.

c LGR: logistic regression.

d DT: decision tree.

e RF: random forest.

f XGB: extreme gradient boosting.

g CAT: CatBoost

h LightGBM: light gradient boosting machine

i ROSE: random over sampling example.

j This method's model results have the highest AUC and a small standard deviation, indicating a relatively good ability to make judgments.

### Performance Estimation and Statistical Testing

Within each dataset, model performance was summarized from 5-fold cross-validation. For each metric (AUC, accuracy, and *F*_1_-score), we calculated 95% CIs from the 5-fold–specific estimates using a 2-sided Student t interval. This procedure corresponds to applying the *t.test* function in R to the vector of fold-level values. Because these metrics are bounded between 0 and 1, upper limits were truncated at 1.0 when necessary, and models with zero variance across folds yield a point interval at the mean.

For pairwise AUC comparisons within the same dataset, we applied the nonparametric DeLong test to out-of-fold predictions pooled across the 5 validation folds, using the *roc.test* function from the *pROC* package. We report 2-sided *P* values; all tests are conducted within the dataset, and we do not compute pooled AUCs or conduct between-dataset hypothesis tests.

### Model Refinement and Variable Selection-Phase 2

In the second phase, the AUC [36] was used to evaluate the models from the first phase. For the optimal data processing method and the corresponding best-performing ML model, a detailed scoring and ranking of variables was conducted based on their importance and contribution to the model’s predictive performance.

The variables were converted into percentile rankings, with the most important variable assigned a score of 100, while the least important variable was assigned a score of 0. Using the 5-fold cross-validation approach, the scores from 5 iterations were summed and ranked, with the highest possible score being 500. Based on this scoring system, the top 15 highest-ranked variables were selected for remodeling, allowing for a more focused and in-depth analysis of their impact on the outcomes.

### Ethical Considerations

This study was reviewed and approved by the Institutional Review Board of Shin-Kong Wu Ho-Su Memorial Hospital (IRB No. 20231101R; approval date: December 14, 2023). The requirement for informed consent was waived by the ethics committee due to the retrospective nature of the study and the use of deidentified data. All procedures were conducted in accordance with the ethical standards of the responsible institutional and national committees on human experimentation and with the principles of the Declaration of Helsinki. The privacy and confidentiality of all participants were strictly protected throughout the study, and no personally identifiable information was disclosed. No compensation was provided to participants.

## Results

### Phase 1

In this phase, we systematically evaluated the predictive performance of multiple ML models, including logistic regression, decision trees, random forests, XGBoost, CatBoost, and LightGBM, using 5-fold cross-validation. The primary objective was to determine the most effective combination of encoding methods (one-hot Encoding, frequency encoding, and target encoding) and data imbalance handling techniques (ROSE and SMOTE) for predicting MACE.

Models were evaluated by accuracy, *F*_1_-score, and AUC with 5-fold cross-validation, and Table 2 reports the fold mean, SD, and t-distribution–based 95% CIs for each combination. Among all pipelines, the OneHotE_ROSE–LightGBM model achieved the best overall performance, with mean accuracy of 0.932 (SD 0.112; 95% CI 0.759‐1.000), *F*_1_-score of 0.918 (SD 0.137; 95% CI 0.754‐1.000), and AUC of 0.940 (SD 0.116; 95% CI 0.794‐1.000). Frequency and target encoding under ROSE also performed strongly (AUC 0.913 and 0.900, respectively), but at a slightly lower level than one-hot encoding in the same setting.

**Figure 3.**
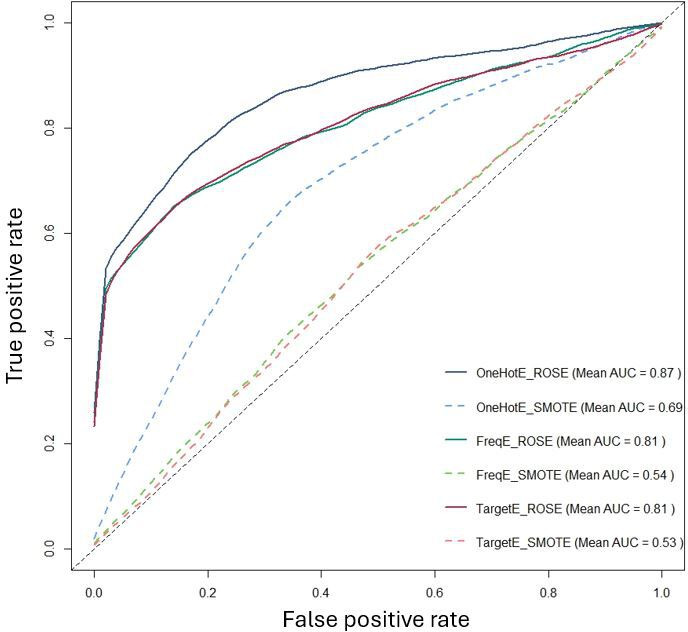
Comparison of the average area under the ROC curve (AUC) of different data models. AUC: area under the ROC curve; ROSE: Random Over Sampling Example; SMOTE: Synthetic Minority Over-sampling Technique.

To formally assess differences between preprocessing strategies, we performed pairwise DeLong tests on out-of-fold AUCs, with results summarized in Table S2 in Multimedia Appendix 1. Most ROSE pipelines showed significantly higher AUC than their SMOTE counterparts (all *P*≤.002), and OneHotE_ROSE in particular was markedly superior to OneHotE_SMOTE. Within the ROSE group, OneHotE_ROSE, FreqE_ROSE, and TargetE_ROSE did not differ significantly from each other (*P*≥.67), indicating a top-performing cluster. Together with the averaged ROC curves in Figures3^5^, these results support selecting one-hot encoding with ROSE as the primary preprocessing strategy for Phase 2.

**Figure 4.**
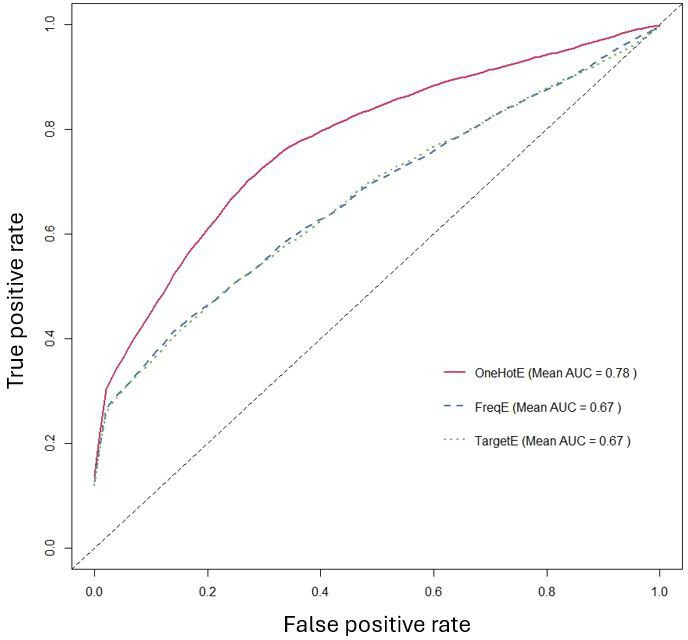
Comparison of the average area under the ROC curve (AUC) of different encoding methods. AUC: area under the ROC curve.

**Figure 5.**
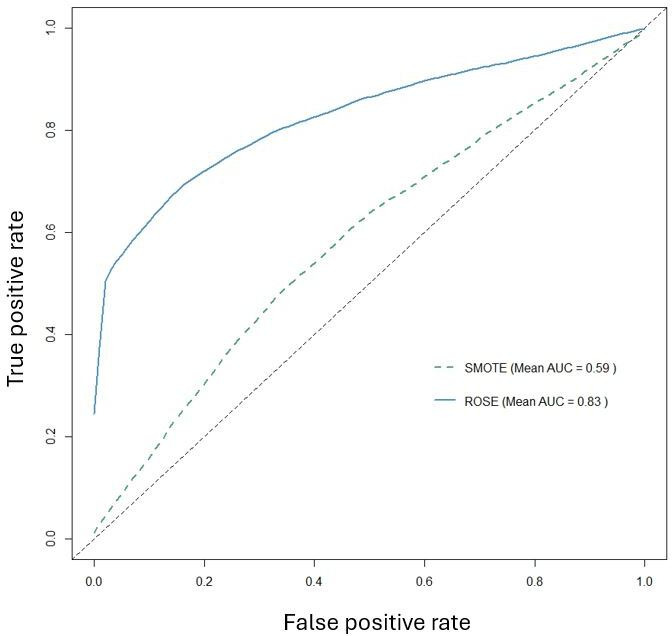
Comparison of the average area under the ROC curve (AUC) of different imbalance methods. AUC: area under the ROC curve; ROSE: Random Over Sampling Example; SMOTE: Synthetic Minority Over-sampling Technique.

A direct comparison of encoding methods in Figure 4 further confirmed that one-hot encoding was the most effective approach, achieving the highest average AUC (0.78), outperforming both frequency encoding and target encoding (both at 0.67). Meanwhile, Figure 5 illustrates that ROSE significantly outperformed SMOTE, achieving an average AUC of 0.83, compared to 0.59 for SMOTE. This result reinforces the conclusion that ROSE generates more representative synthetic samples, preserves the original data distribution more effectively, and enhances model generalization.

Our findings strongly suggest that one-hot encoding combined with ROSE is the most effective preprocessing strategy for this predictive task. This combination preserves categorical feature integrity while mitigating class imbalance, leading to the most robust and accurate predictive models. In contrast, SMOTE not only struggled to enhance model performance but, in some cases, even contributed to its degradation. These insights guided the selection of the optimal model for further feature importance analysis in Phase 2.

### Phase 2

Following the identification of the optimal preprocessing strategy in Phase 1, we conducted a detailed analysis of feature importance and remodeled the dataset using the most influential variables. Table 4 presents the top 15 most important variables ranked based on their cumulative scores from 5-fold cross-validation, with antiplatelet, chest X-ray for aortic arch calcification (CXR.AoAC).0, and insulin emerging as the most significant predictors. These variables were selected to refine the models, ensuring a more efficient and interpretable predictive framework.

**Table 4.** The top 15 variables ranked by importance after 5-fold cross-validation.

Ranking	Feature	Score^a^
1	Antiplatelet	496
2	CXR.AoAC^b^.0	466
3	Insulin	453
4	No.of.hypotension.episodes	435
5	CXR.AoAC.2	434
6	Dyslipid	410
7	DM^c^	392
8	AVA^d^	378
9	AR^e^.0	367
10	AV.cal^f^	348
11	PAOD^g^	337
12	LVH^h^.type.1	302
13	Calcitriol	299
14	AS^i^.0	295
15	Statin	292

a The score calculation method is as follows: in the 5-fold cross-validation, the fields are sorted according to their importance, with the highest being 100 and the lowest being 0. The scores from the 5 folds are then summed to obtain the final score.

b CXR_AoAC: chest X-ray for aortic arch calcification.

c DM: diabetes mellitus.

d AVA: aortic valve area.

e AR: aortic regurgitation.

f AV cal: aortic valve calcification.

g PAOD: peripheral arterial occlusive disease.

h LVH: left ventricular hypertrophy.

i AS: aortic stenosis.

The high-performing ML models from Phase 2 identified a set of correlated features predictive of MACE in patients with ESRD, which can be grouped into 3 main clusters, including structural predictors of MACE, systemic burdens with traditional risk factors, and other indicators of disease severity. The corresponding feature importance profile for the Phase 2 LightGBM model is visualized in Figure S1 in Multimedia Appendix 1. The first group includes aortic arch calcification statuses (CXR.AoAC.0 and CXR.AoAC.2), and concentric LVH (LVH.type.1). These features reflect the model’s ability to capture structural markers representative of the broader spectrum of chronic kidney disease–mineral and bone disorder pathology. Moderate AoAC and AV calcification signal cumulative metabolic and inflammatory insults from longstanding phosphate imbalance, high uremic toxin burden, and chronic inflammation [37-39], while the absence of calcification indicates a protective cardiovascular profile to MACE risk. Additionally, concentric LVH has been long recognized as an independent predictor of MACE and all-cause mortality in patients with ESRD [17,40], results from chronic pressure overload, often due to arterial stiffness secondary to vascular calcification. Collectively, these structural changes contribute to hemodynamic consequences such as heart failure and fatal arrhythmias, thereby resulting in higher MACE risk [41].

The second group of predictors comprises DM, dyslipidemia, and PAOD. DM and dyslipidemia are well-established contributors to atherosclerosis and are exacerbated in ESRD [42,43], forming a vicious cycle with both ESRD-specific and traditional risk factors. For example, hyperglycemia promotes the accumulation of advanced glycation end-products due to impaired clearance, leading to endothelial dysfunction, oxidative stress, and inflammation, thereby fueling the atherosclerosis process [44]. PAOD, a direct manifestation of exacerbated systemic atherosclerosis, also shares similar underlying pathophysiology. Despite not being an isolated predictor, PAOD’s strong association to higher MACE risk [45] makes it a valuable proxy for assessing a patient’s total atherosclerotic burden.

The third group includes antiplatelet, intradialytic hypotension (IDH), and valvular disease status. With a score of 496, antiplatelet use serves as a surrogate marker for clinician-identified ischemic risk and thus MACE vulnerability [46]. Furthermore, the number of IDH episodes ranked fourth, underscoring the hemodynamic stress of dialysis, with cumulative episodes contributing to myocardial stunning and ischemic damage, thus in turn linked to MACE [47]. Tracking IDH frequency could offer a more comprehensive view of cardiovascular stress rather than focusing on isolated episodes as well as the episode severity. In addition, the absence of valvular diseases such as AS or aortic regurgitation reflects a mitigated risk of LVH and atherosclerosis progression [48]. The Phase 2 model effectively learned patterns of clinical practice—such as medication use and hemodynamic instability—offering real-world applicability and insights that extend beyond static risk markers to isolated hypotensive events.

The models were then retrained with one-hot encoding combined with either ROSE or SMOTE, and their performance was evaluated by accuracy, *F*_1_-score, and AUC with 5-fold cross-validation (Table 3). Among the ROSE-based models, LightGBM and XGBoost achieved the highest discrimination, with LightGBM reaching accuracy 0.906 (SD 0.115; 95% CI 0.763‐1.000), *F*_1_-score 0.892 (SD 0.119; 95% CI 0.745‐1.000), and AUC 0.925 (SD 0.112; 95% CI 0.786‐1.000), and XGBoost reaching accuracy 0.913 (SD 0.122; 95% CI 0.762‐1.000), *F*_1_-score 0.899 (SD 0.138; 95% CI 0.728‐1.000), and AUC 0.906 (SD 0.137; 95% CI 0.736‐1.000). Random forest under ROSE also performed well (AUC 0.915; 95% CI 0.750‐1.000), confirming that ensemble methods benefited most from the refined feature set.

To place these gains in context, we also examined the traditional logistic regression (LGR) baseline in the same table. LGR trained in only 0.08 s for both ROSE and SMOTE variants, confirming its computational efficiency. However, its discrimination lagged behind the ML models, with AUC values of 0.755 (ROSE) and 0.728 (SMOTE). This contrast highlights a clear trade-off: while LGR offers near-instantaneous runtime, the proposed ML pipeline delivers markedly stronger predictive accuracy, justifying its added complexity for clinical applications.

To validate the statistical significance of these differences, we conducted DeLong tests for all model pairs, with detailed *P* values reported in Table S3 in Multimedia Appendix 1. ROSE-based ensembles (LightGBM, XGBoost, and random forest) achieved significantly higher AUCs than all SMOTE-based models (all *P*≤.022), while differences among the ROSE ensembles themselves were small and often not statistically significant. In contrast, the logistic regression baseline under ROSE achieved AUC 0.755 (SD 0.050; 95% CI 0.694‐0.817), clearly lagging behind the gradient boosting models despite its very short training time. These findings confirm that combining ROSE with gradient-boosted ensembles yields the most stable and discriminative performance in the Phase 2 setting.

These findings highlight the robustness of ROSE in preserving critical data characteristics, while the selection of the top 15 features enhanced model efficiency without compromising predictive accuracy. The combination of ROSE with XGBoost and LightGBM yielded the most reliable performance, reinforcing the effectiveness of targeted feature selection and imbalance handling in MACE prediction.

## Discussion

### Principal Results

**Figure 6.**
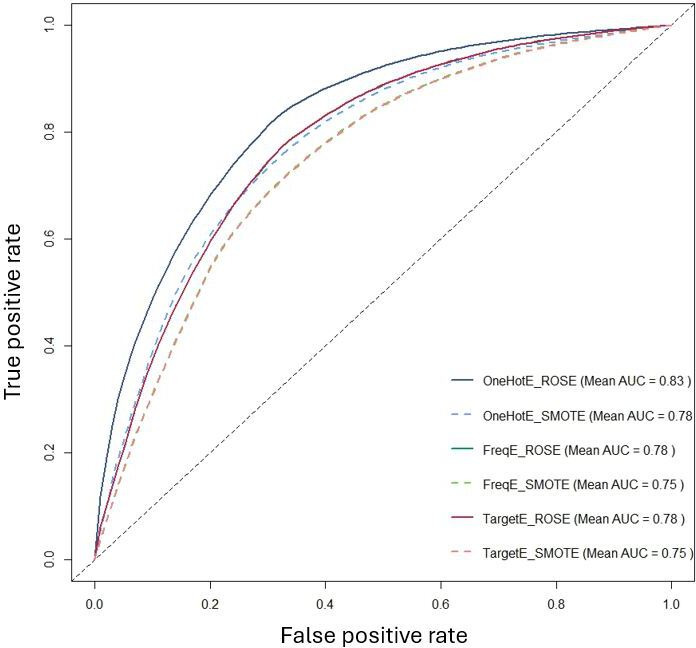
Comparison of the average area under the ROC curve (AUC) of different data models. AUC: area under the ROC curve; ROSE: Random Over Sampling Example; SMOTE: Synthetic Minority Over-sampling Technique.

To assess cross-dataset portability and reproducibility, we applied the integrated preprocessing and modeling framework to 2 datasets with distinct data-generating mechanisms: an in-house ESRD registry (n=412) and the population-based BRFSS 2015 survey. Cross-dataset comparisons of model performance, encoding strategies, and imbalance-handling methods are summarized in Figures6^8^. For each dataset, the full workflow was executed independently, including missForest imputation, one-hot, frequency, target encoding, ROSE or SMOTE resampling, model tuning, and evaluation, with no cross-dataset feature matching and no pooled AUC computations. The framework yielded improved discrimination and clinically interpretable feature-importance rankings in both settings.

**Figure 7.**
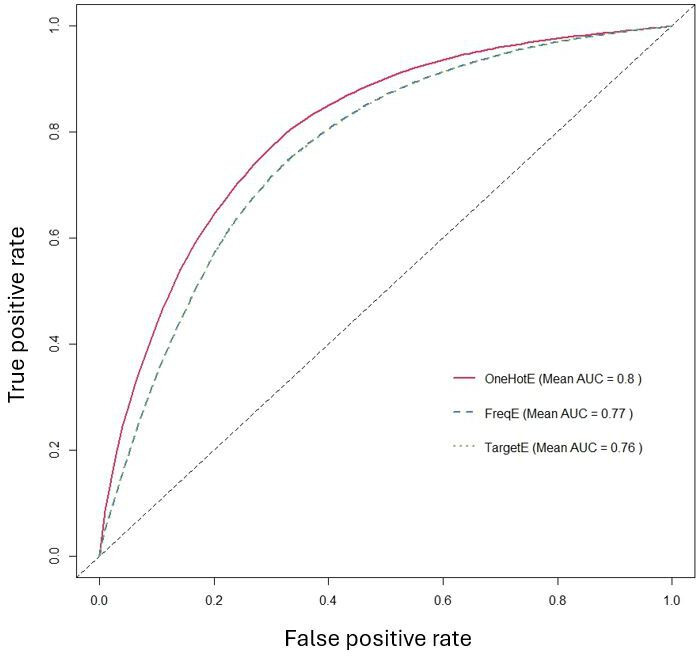
Comparison of the average area under the ROC curve (AUC) of different encoding methods. AUC: area under the ROC curve; ROSE: Random Over Sampling Example; SMOTE: Synthetic Minority Over-sampling Technique.

**Figure 8.**
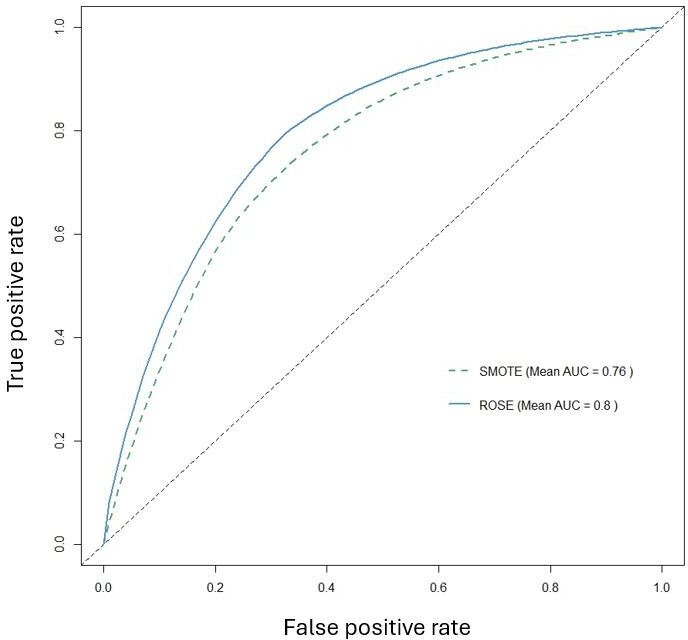
Comparison of the average area under the ROC curve (AUC) of different imbalance methods. AUC: area under the ROC curve; ROSE: Random Over Sampling Example; SMOTE: Synthetic Minority Over-sampling Technique.

### Limitations

Our study has several constraints that warrant consideration. The ESRD cohort includes 412 patients, which limits the capture of rare patterns; multicenter expansion is ongoing to increase diversity. Despite 5-fold cross-validation, nested grid search, and an independent BRFSS run to assess cross-dataset portability, overfitting cannot be fully excluded, so temporal and multi-site validations are planned. Although the workflow performed well for cardiovascular and metabolic endpoints, its applicability to other disease domains remains uncertain and will be explored. By design, the BRFSS analysis did not involve feature harmonization, pooled AUCs, or between-dataset hypothesis tests. Finally, while ROSE outperformed SMOTE in our experiments, optimal resampling and encoding choices are likely dataset dependent, and hybrid strategies and embedding-based representations merit further investigation.

### Comparison With Prior Work

In contrast to previous studies that tend to focus on isolated aspects of data preprocessing, our research offers a comprehensive, end-to-end framework that addresses the multifaceted challenges inherent in clinical data, namely high missingness, heterogeneity, and severe class imbalance. While earlier works typically investigated single techniques in a limited scope, our study integrates multiple advanced methods and validates the entire pipeline using cross-dataset comparisons. This holistic approach provides a stronger foundation for both improving predictive performance and facilitating clinical translation, marking a clear advancement over traditional, piecemeal methodologies.

### Conclusions

This study introduces a robust, generalizable, and interpretable data preprocessing framework for predicting MACEs among patients with ESRD. Although the improvements in performance metrics are incremental, they directly address key challenges inherent in clinical data heterogeneity and quality. The proposed pipeline enhances both predictive reliability and model transparency, offering practical value for clinical decision support and operational planning in nephrology care.

While system-level integration was not the primary focus of this work, the pipeline was deliberately designed for seamless adoption in real-world settings. By using routinely available clinical variables and lightweight computational procedures, it can be incorporated into existing analytics environments with minimal architectural adjustment. This design supports efficient deployment across diverse institutions and datasets, as evidenced by the consistent performance observed in both the ESRD registry and the BRFSS dataset. Such scalability reinforces its translational potential for broader clinical and public health applications.

Future work should prioritize cross-context application across more heterogeneous populations and evaluate the integration of deep learning architectures to further enhance predictive accuracy and extend applicability to other high-risk patient groups and chronic disease domains.

## Supplementary material

10.2196/75655 Multimedia Appendix 1 Supplementary tables and figures including hyperparameter grid search settings, DeLong test *P* value matrices, dataset-specific SMOTE/ROSE settings and random seeds, LightGBM feature importance, and the leakage-safe cross-validation workflow.

## Abbreviations

AS: aortic stenosis
AUC: area under the ROC curve
BRFSS: Behavioral Risk Factor Surveillance System
CAT: CatBoost
CXR_AoAC: chest X-ray for aortic arch calcification
DM: diabetes mellitus
DT: decision tree
ESRD: end-stage renal disease
IDH: intradialytic hypotension
LGR: logistic regression
LightGBM: light gradient-boosting machine
LVH: left ventricular hypertrophy
MACE: Major Adverse Cardiovascular Events
ML: machine learning
OOF: out-of-fold
PAOD: peripheral arterial occlusive disease
ROS: Random Over-Sampling
ROSE: Random Over-Sampling Examples
SMOTE: Synthetic Minority Over-sampling Technique
XGB: extreme gradient boosting (XGBoost)
