# Supplementary material for: Enhancing Model Generalizability in Medical Artificial Intelligence: Systematic Comparison of Categorical Encoding and Sampling Techniques for Imbalanced Data
Source: JMIR Med Inform. 2026 Apr 13;14:e75655. doi: 10.2196/75655 (PMC13075634; doi:10.2196/75655)
Supplement: Multimedia Appendix 1 [file medinform-v14-e75655-s001.docx]

## Multimedia Appendix 1. Supplementary tables and figures including hyperparameter grid search settings, DeLong test p-value matrices, dataset-specific SMOTE/ROSE settings and random seeds, LightGBM feature importance, and the leakage-safe cross-validation workflow.

Table S1: Grid search Key Hyper‑parameters

| **Model** | **Key Hyper‑parameters** | **Candidate Values** |
| --- | --- | --- |
| CatBoost | depth | 6 , 8 , 10 |
|  | learning_rate | 0.01 , 0.03 , 0.1 |
|  | l2_leaf_reg | 2 , 3 , 5 |
|  | iterations | 800 , 1000 |
| LightGBM | num_leaves | 10 , 20 , 30 |
|  | learning_rate | 0.05 , 0.1 , 0.2 |
|  | num_iterations | 800 , 1000 , 3000 |
| RandomForest | mtry | $\sqrt{p}$,  p/3 |
|  | ntree | 200 , 500 |
|  | nodesize | 1 , 5 |
|  | replace | TRUE |
| XGBoost | eta | 0.1 , 0.2 |
|  | max_depth | 4 , 6 |
|  | nrounds* | 1000 |
| DecisionTree | cp | 0.001 , 0.01 , 0.1 |
|  | minsplit | 10 , 20 , 30 |
|  | maxdepth | 5 , 10 , 15 |
| Logistic Reg. | — | baseline |

Table S2: Phase 1 Delong test P- value matrix

|  | ONE HOT_ SMOTE | ONE HOT_ ROSE | Frequency_ SMOTE | Frequency_ ROSE | Target_ SMOTE | Target_ ROSE |
| --- | --- | --- | --- | --- | --- | --- |
| ONE HOT_ SMOTE | 1.0000 | 0.0000 | 0.0002 | 0.0000 | 0.0002 | 0.0000 |
| ONE HOT_ ROSE | 0.0000 | 1.0000 | 0.0000 | 0.7738 | 0.0000 | 0.6686 |
| Frequency_ SMOTE | 0.0002 | 0.0000 | 1.0000 | 0.0000 | 1.0000 | 0.0000 |
| Frequency_ ROSE | 0.0000 | 0.7738 | 0.0000 | 1.0000 | 0.0000 | 1.0000 |
| Target_ SMOTE | 0.0002 | 0.0000 | 1.0000 | 0.0000 | 1.0000 | 0.0000 |
| Target_ ROSE | 0.0000 | 0.6686 | 0.0000 | 1.0000 | 0.0000 | 1.0000 |

Table S3: Phase 2 One Hot Encoding Delong test P- value martix

|  | SMOTE CAT | SMOTE LightGBM | SMOTE RF | SMOTE XGB | SMOTE DT | SMOTE LGR | ROSE CAT | ROSE LightGBM | ROSE RF | ROSE XGB | ROSE DT | ROSE LGR |
| --- | --- | --- | --- | --- | --- | --- | --- | --- | --- | --- | --- | --- |
| SMOTE CAT | 1 | 0.8133 | 0.4093 | 0.74 | 0.7104 | 0.6378 | 0 | 0 | 0 | 0 | 0.0013 | 0.0219 |
| SMOTE LightGBM | 0.8133 | 1 | 0.2487 | 0.912 | 0.9374 | 0.4676 | 0 | 0 | 0 | 0 | 0.0005 | 0.0091 |
| SMOTE RF | 0.4093 | 0.2487 | 1 | 0.0619 | 0.2466 | 0.7589 | 0 | 0 | 0 | 0 | 0.0024 | 0.0554 |
| SMOTE XGB | 0.74 | 0.912 | 0.0619 | 1 | 0.9875 | 0.4036 | 0 | 0 | 0 | 0 | 0.0002 | 0.0097 |
| SMOTE DT | 0.7104 | 0.9374 | 0.2466 | 0.9875 | 1 | 0.3547 | 0 | 0 | 0 | 0 | 0.0005 | 0.0062 |
| SMOTE LGR | 0.6378 | 0.4676 | 0.7589 | 0.4036 | 0.3547 | 1 | 0 | 0 | 0 | 0 | 0.002 | 0.0014 |
| ROSE CAT | 0 | 0 | 0 | 0 | 0 | 0 | 1 | 0.1963 | 0.0011 | 0 | 0 | 0 |
| ROSE LightGBM | 0 | 0 | 0 | 0 | 0 | 0 | 0.1963 | 1 | 0.0676 | 0 | 0 | 0 |
| ROSE RF | 0 | 0 | 0 | 0 | 0 | 0 | 0.0011 | 0.0676 | 1 | 0 | 0 | 0 |
| ROSE XGB | 0 | 0 | 0 | 0 | 0 | 0 | 0 | 0 | 0 | 1 | 0 | 0 |
| ROSE DT | 0.0013 | 0.0005 | 0.0024 | 0.0002 | 0.0005 | 0.002 | 0 | 0 | 0 | 0 | 1 | 0.0864 |
| ROSE LGR | 0.0219 | 0.0091 | 0.0554 | 0.0097 | 0.0062 | 0.0014 | 0 | 0 | 0 | 0 | 0.0864 | 1 |

Table S4: Dataset-specific SMOTE and ROSE parameter settings and random seeds

| Data | Imbalance | method | seed |
| --- | --- | --- | --- |
| SKH_ESRD | SMOTE | dist = HEOM, C.perc =list("0" = 1, "1"= 1.423) | 123 |
|  | ROSE | method = over, p = 0.5 | 123 |
| BRFSS2015 | SMOTE | dist = HEOM, C.perc =list("0" = 1, "1"= 6.177) | 123 |
|  | ROSE | method = over, p = 0.5 | 123 |


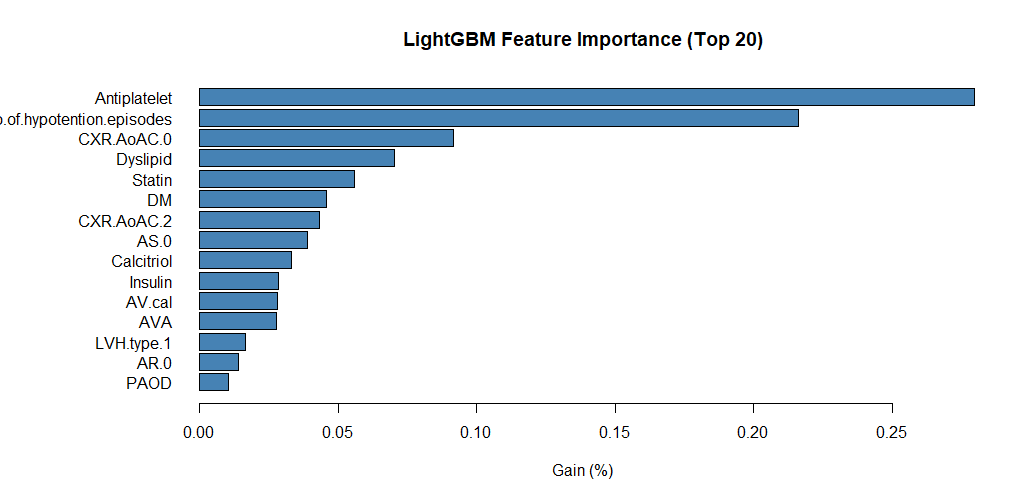


Figure S1 Phase 2 LightGBM Model importance

Algorithm S1: Leakage-safe workflow with K-fold CV and re-fit

**Inputs**:

Datasets {ESRD, BRFSS}; split ratio 80:20; K=5

Imputer: missForest

Encoders: One-hot, Frequency, Target (OOF smoothing)

Samplers: ROSE, SMOTE

Models: {LGR, DT, RF, XGB, LGBM, CAT}

**Output**:

OOF predictions, CV metrics, held-out Test metrics, selected features, seeds

**for** each dataset **D** in {ESRD, BRFSS}:

1) Split

Stratified split **D** into Train 80% and Test 20% with fixed seed.

2) Primary CV loop on Train (feature screening and model selection are done here)

**for** each pipeline p in {Imputer → Encoder → Sampler → Model}:

Initialize OOF := NaN

**for** fold k in 1..K:

($train_{k}, val_{k}$) := k_th_ stratified split of Train

# Impute

$Imputer_{k}$ := fit on train_k

$trai{n_{k}}_{imp}$ := transform Imputer_k_(train_k_)

$va{l_{k}}_{imp}$ := transform Imputer_k_(val_k_)

# Encode

**if** Encoder is Target:

$trai{n_{k}}_{enc}$ := inner OOF target encoding on $trai{n_{k}}_{imp}$

$va{l_{k}}_{enc}$ := apply target stats from $trai{n_{k}}_{imp}$ to $va{l_{k}}_{imp}$

**else**:

$Encoder_{k}$ := fit on train_k_imp

$trai{n_{k}}_{enc}$ := transform Encoder_k_($trai{n_{k}}_{imp}$)

$va{l_{k}}_{enc}$ := transform Encoder_k_($va{l_{k}}_{imp}$)

# Rebalance training only

$trai{n_{k}}_{bal} , {r_{params}}_{k}$ := apply Sampler to $trai{n_{k}}_{enc}$ (target ratio 1:1)

# $val_{k}$ remains untouched

# Train and validate

$\theta_{k}$ := tune Model on $trai{n_{k}}_{bal}$ using inner CV or holdout inside $train_{k}$

$f_{k}$ := fit Model with $\theta_{k}$ on $trai{n_{k}}_{bal}$

OOF[$va{l_{idx}}_{k}$] := predict_proba $f_{k}$($va{l_{k}}_{enc}$)

record per-fold metrics and ${r_{params}}_{k}$

**end for**

$C{V_{metrics}}_{p}$ := aggregate per-fold metrics

store OOF_p_ and $C{V_{metrics}}_{p}$

**end for**

3) Feature screening on Train

Use OOF predictions and model-based importance computed within CV

Select a stable feature set S* according to a predeclared rule

4) Second-stage modeling on Train with S*

Re-run Step 2 only for features S* to finalize the best pipeline p* and θ*

5) Final refit on full Train and evaluation on Test

Fit Imputer on full Train; transform Train and Test

Fit Encoder on full Train; transform Train and Test

Apply Sampler to Train only

Fit Model p* with θ* on rebalanced Train

Test_metrics := evaluate on untouched Test

Save OOF, $CV_{metrics}$ of p*, $Test_{metrics}$, selected features S*, θ*, seeds, and resampling parameters.

**Notes**:

-ESRD and BRFSS are processed independently end to end.

-Target encoding uses out-of-fold smoothing inside Train folds.

-Validation and Test are never resampled.

-No cross-dataset feature matching. No pooled AUC or between-dataset hypothesis tests
